# Morphological and topographic analysis of the sesamoid bones of the hand: A radiographic institution-based descriptive cross-sectional study

**DOI:** 10.1016/j.jpra.2025.10.017

**Published:** 2025-10-16

**Authors:** Phajir V. Santosh Rai, Ravichandraprabhu Abisshek Balaji, Madhvi Yadav, Jefferson Prince, Latha V. Prabhu, Rohini Punja, Mamatha Hosapatna, Bukkambudhi V. Murlimanju

**Affiliations:** a Department of Radiodiagnosis, Kasturba Medical College Mangalore, Manipal Academy of Higher Education, Manipal, India; b Department of General Surgery, Kasturba Medical College Mangalore, Manipal Academy of Higher Education, Manipal, India; c Department of Anatomy, Kasturba Medical College Mangalore, Manipal Academy of Higher Education, Manipal, India; d Department of Oral and Maxillofacial Surgery, Manipal College of Dental Sciences Mangalore, Manipal Academy of Higher Education, Manipal, India; e Department of Anatomy, Kasturba Medical College, Manipal Academy of Higher Education, Manipal, India

**Keywords:** Hand, Osteoarthritis, Radiographic film, SDG-3, Sesamoid bones

## Abstract

The goal of this study was to establish the incidence, topographic location and measurements of sesamoid bones of the hand. This cross-sectional study involved 102 antero-posterior plain radiographs of the hand. Among them, 56 were female patients, and 46 were male patients. The films were scrutinized for their topography and number of sesamoid bones. Radiant Dicom viewer software (Poland, version 4.2.1.17555, 64-bit) was used to determine morphometric parameters such as the length and breadth of the sesamoid bones. Side, age and sex comparisons of the parameters were performed via the chi-square test. The sesamoid bone was observed at the first metacarpophalangeal joint, medially and laterally, in 99% and 80.6% of the patients, respectively. The length and breadth of the medial sesamoid and lateral sesamoid were 0.49 ± 0.07 cm and 0.40 ± 0.07 cm, 0.52 ± 0.10 cm and 0.37 ± 0.08 cm, respectively. The second, third and fifth metacarpophalangeal joints exhibited a single sesamoid bone, and they were present in 25.5%, 3.1% and 46.9% of the patients, respectively. Their length and breadth were 0.46 ± 0.10 cm and 0.36 ± 0.07 cm, 0.42 ± 0.14 cm and 0.34 ± 0.17 cm, 0.38 ± 0.06 cm and 0.33 ± 0.04 cm, respectively. The sesamoid bones had a greater incidence in females (*p* < 0.05), sidewise and the age wise comparison did not yield a statistically significant difference (*p* > 0.05). The incidence, location, and morphometric data of sesamoid bones of the hand offered by this study can be considered the morphological database for the sample population studied. The data will be enlightening to the operating hand surgeon, plastic surgeon, radiologist and anthropologist.

## Introduction

Sesamoid bones are common in the hand and feet. Five sesamoid bones usually exist in the hand, among which two are observed in the metacarpophalangeal (MCP) joint of the thumb, one at the interphalangeal joint of the thumb, and one each at the MCP joints of the index and little fingers.^1^ However, the presence of more sesamoid bones has also been reported in the literature.^2^ Sesamoid bones are found beneath the tendons that cross the joints, thereby helping in the gliding mechanism by reducing friction as well as modifying pressure.^3^ Usually, sesamoid bones are formed in response to strain to either muscle or tendons.^4^

Although sesamoid bones are easily observed in radiographs, pathologies related to sesamoid bones are commonly missed because of their small size and focus on the larger bones nearby them.^5^ Ossification of the sesamoid bone at the medial aspect of the first MCP joint is considered an indicator of the onset of puberty in males and females.^6^^,^^7^ However, differences in the distribution and prevalence of sesamoid bones among different ethnic groups, ancestral groups and populations have been described.^8^ In a South American study, there was a difference in the prevalence of sesamoid bones between the Chilean and Brazilian sub-populations.^9^ This finding indicates that additional studies across diverse populations are needed to better understand these variations. A literature review did not reveal many studies on sesamoid bones in our sample South Indian population. Since sesamoid bones play crucial roles in biomechanics and range of motion of hand joints, knowledge of their incidence and morphology is important. While hand surgery is performed, prior knowledge is essential, as the presence of these rare sesamoid bones is challenging for surgeons. This study supports the ‘Sustainable Development Goal-3′ (SDG-3), which aims to ensure healthy lives and promote well-being for all, at all ages. Considering the above factors and rationale, the objectives of the present study were to determine the incidence, topography and dimensions of sesamoid bones of the hand in a sample from South India.

## Materials and methods

The present institution-based descriptive cross-sectional study was performed at the Department of Radiodiagnosis of Kasturba Medical College Mangalore, Manipal Academy of Higher Education, Manipal, India. It included 102 antero-posterior view plain radiographs of the hand (51 right side and 51 left side). Among them, 46 (45.1 %) were male, and 56 (54.9 %) were female. The sample size of each study was calculated via a statistical formula that considers the numbers and variables from previous publications. Radiographs were obtained from hospitals from January 2025 to June 2025, which are associated with our teaching institution. The identities of the patients and other details were not disclosed in this study; hence, written informed consent was not needed, as the samples were anonymized. The manuscript has been prepared in accordance with the STROBE guidelines and the checklist of items that should be included in reports of cross-sectional studies. The institutional review board of our college approved this study on 19.12.2018 (IEC KMC MLR 12-18/497). Patients above or at the age of fourteen, South Indian by domicile and with good-quality antero-posterior radiographs revealing all the bones of the hands were the inclusion criteria of this study. Ossification of sesamoid bones in the hand, particularly at the thumb MCP joint, generally occurs around the age of fourteen years; hence, this age group was also included in this study. Radiographs that revealed pathological changes such as fractures, arthritis and calcification; suspected signs and symptoms due to sesamoid bones; and poor-quality radiographs were excluded. The number of sesamoid bones and their exact topography were observed in hand radiographs. The length and breadth of the sesamoid bones were measured via the software “Radiant Dicom Viewer” (Poland, version 4.2.1.17555, 64-bit). The measurements were performed by a single person, and the average of the three repeated measurements was considered. This approach was used to prevent intraobserver and interobserver bias. This study aimed to analyze the factors that influence the number of sesamoid bones in the hand. The factors considered were the sex of the sample, age of the sample, sidewise comparison and number of digits with the most sesamoid bones. The data were analyzed via the recent version of SPSS software, and the chi square test was applied for age, sex and side-based comparisons. The “*p*” value was considered significant if it was less than 0.05.

## Results

**Figure 1:**
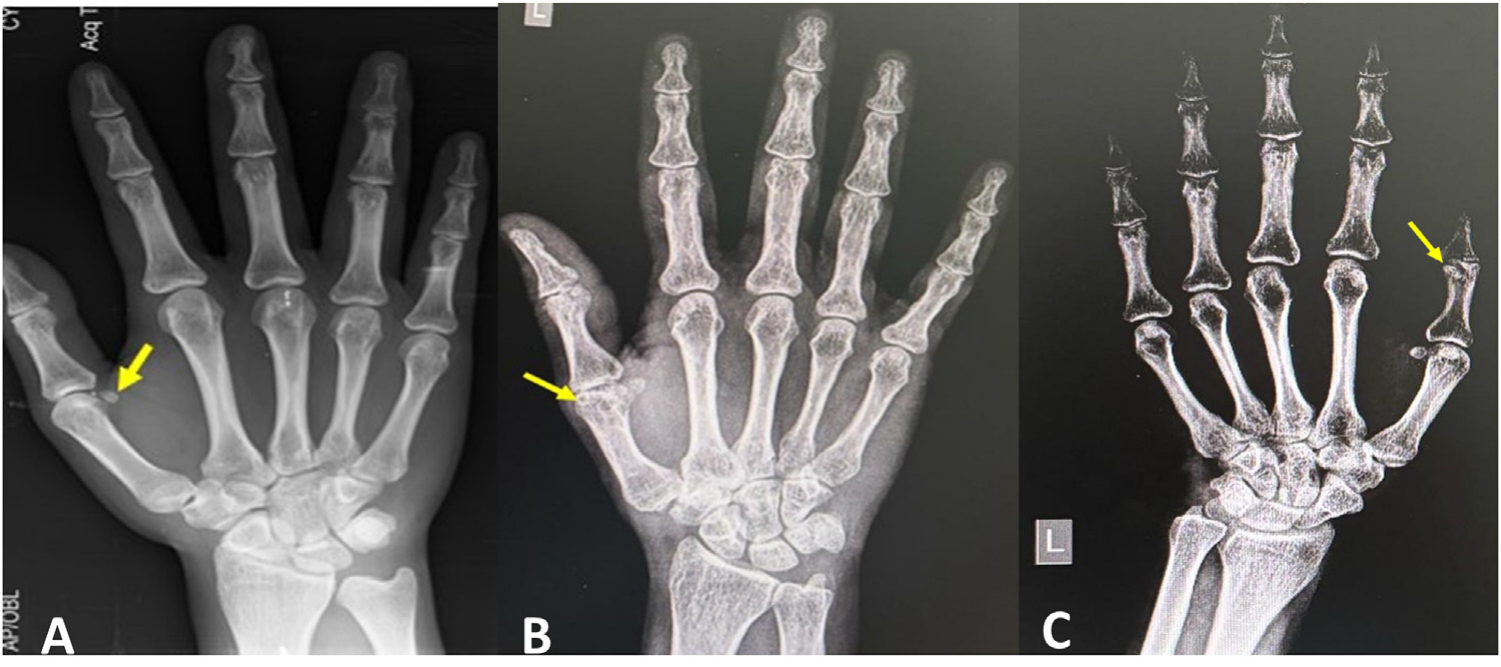
Plain radiographs (anteroposterior views) of the hand showing the sesamoid bones (marked by the arrows), which are observed at the thumb (first digit); (A) sesamoid bone at the medial aspect of the first metacarpophalangeal joint; (B) sesamoid bone at the lateral aspect of the first carpometacarpal joint; (C). sesamoid bone at the interphalangeal joint.

**Figure 2:**
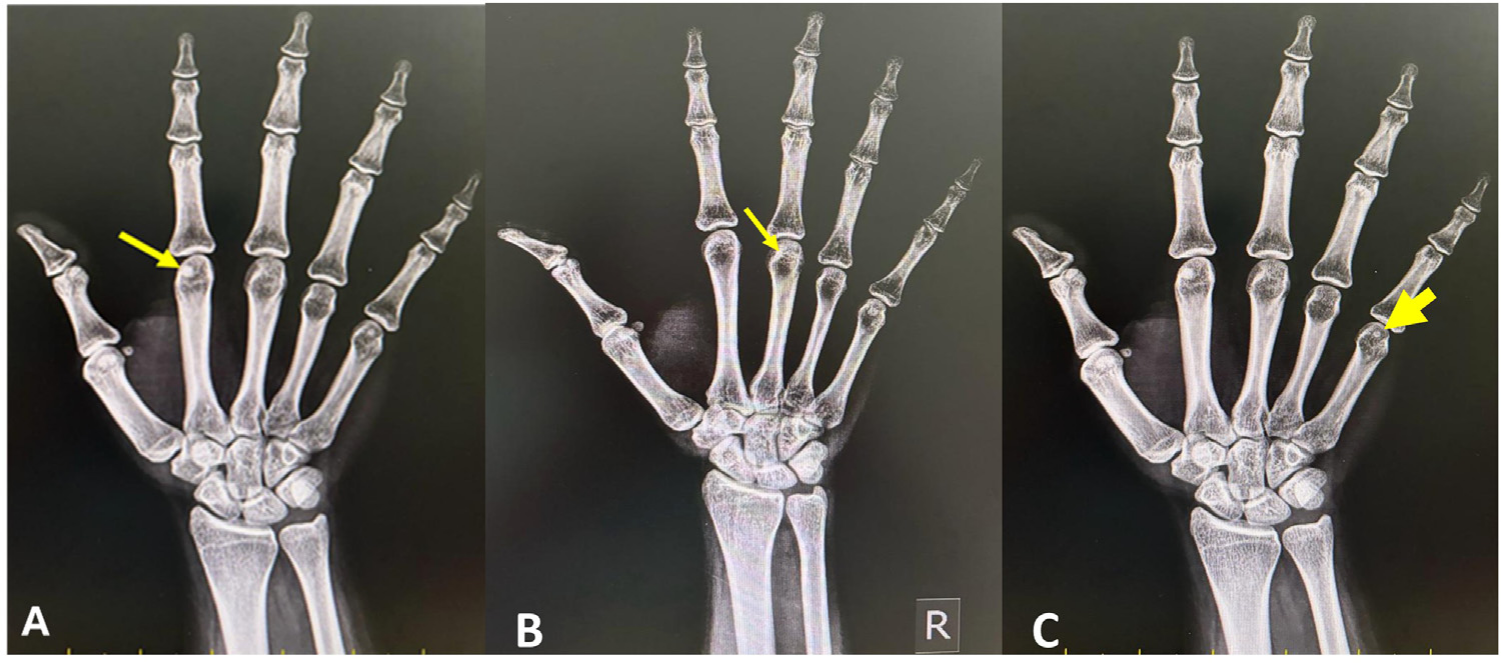
Plain radiographs (anteroposterior views) of the hand showing the sesamoid bones (marked by arrows) at the metacarpophalangeal joints of the second digit (A), third digit (B) and fifth digit (C).

One hundred two antero-posterior view plain radiographs were confirmed to be eligible for the analysis in this study. The incidence of sesamoid bones at each finger of the hand (*n* = 102) and their dimensions are summarized in Table 1. Figure 1, Figure 2 are plain radiographs (anteroposterior views) of the hand, which reveal the sesamoid bones. The sesamoid bone was present at both the medial (100 % of cases, Figure 1A) and lateral aspects (85.3 % of cases, Figure 1B) of the first MCP joint of the thumb. Figure 1C shows the sesamoid bone at the interphalangeal joint (in 47.1 % of the patients) of the thumb. The sesamoid bone at the second (Figure 2A) MCP joint was observed in 25.5 % of the patients. The third MCP joint had a sesamoid bone (Figure 2B) in only 3.1 % of the patients. It was not observed in the fourth MCP joint (0 %). The fifth MCP had a sesamoid bone in 46.9 % of the patients (Figure 2C).

**Table 1:** Incidence and dimensions of sesamoid bones at each finger.

Finger/joint	Medial/lateral	Incidence	Length	Breadth
Thumb MCP	medial	102 (100 %)	0.49 ± 0.08	0.4 ± 0.07
Thumb MCP	lateral	87 (85.3 %)	0.52 ± 0.1	0.37 ± 0.08
IP	–	48 (47.1 %)	0.41 ± 0.1	0.57 ± 0.12
Index finger MCP	–	29 (28.4 %)	0.46 ± 0.1	0.37 ± 0.07
Middle finger MCP	–	2 (2 %)	0.37 ± 0.14	0.29 ± 0.2
Ring finger MCP	–	nil (0 %)		
Little finger	–	48 (47.1 %)	0.38 ± 0.06	0.33 ± 0.05

MCP-metacarpophalangeal joint; IP-interphalangeal joint, measurements in centimeters ± standard deviation.

The incidence of the breakdown of the number of sesamoid bones in the hand and the sex crosstabulation are presented in Table 2. There was a significant difference in the number of sesamoid bones and sex (Table 3). The statistical analysis revealed that females had a greater number of sesamoid bones than males did (*p* = 0.025). The age-related crosstabulation of the number of sesamoid bones in each hand is summarized in Table 4. The correlation between the age of the sample and the number of sesamoids was not statistically significant (*p* = 0.442), as shown in Table 5. The side-based comparison of sesamoid bones in each digit is presented in Table 6. In the fourth digit, the sesamoid bone was not observed on either side. Overall, the side-based comparison of the digits did not reveal a statistically significant difference (*p* > 0.05) in the number of sesamoid bones (Table 6).

**Table 2:** Overall incidence and sex crosstabulation of number of sesamoid bones in the hand (*n* = 102).

Number of sesamoid bones	Overall incidence and percentage	Male	Female
0	nil (0 %)		
1	6 (5.9 %)	2 (1.96 %)	4 (3.92 %)
2	26 (25.5 %)	19 (18.63 %)	7 (6.86 %)
3	38 (37.25 %)	14 (13.72 %)	24 (23.53 %)
4	17 (16.7 %)	6 (5.9 %)	11 (10.78 %)
5	15 (14.7 %)	5 (4.9 %)	10 (9.8 %)

**Table 3:** Chi-Square test analysis of the sex crosstabulation of number of sesamoid bones.

	Value	df	Asymptotic significance (2-sided)
Pearson chi-square	11.100^a^	4	0.025
Likelihood ratio	11.306	4	0.023
Linear-by-linear Association	3.875	1	0.049
Number of valid cases	102		

a Two cells (20 %) have expected count less than 5. The minimum expected count is 2.71.

**Table 4:** Age wise crosstabulation of number of sesamoid bones in each hand (*n* = 102).

Number of sesamoid bones	<18 years (*n* = 5)	18–35 years (*n* = 33)	36–55 years (*n* = 37)	>55 years (*n* = 27)
0	nil (0 %)			
1	1 (1 %)	3 (2.9 %)	nil (0 %)	2 (2 %)
2	1 (1 %)	10 (9.8 %)	7 (6.9 %)	8 (7.8 %)
3	2 (2 %)	11 (10.8 %)	16 (15.7 %)	9 (8.8 %)
4	1 (1 %)	3 (2.9 %)	10 (9.8 %)	3 (2.9 %)
5	nil (0 %)	6 (5.9 %)	4 (3.9 %)	5 (4.9 %)

**Table 5:** Chi-Square test analysis of the age wise crosstabulation of number of sesamoid bones.

	value	df	*p*-value
Pearson chi-square	12.044^a^	12	0.442
Likelihood ratio	14.148	12	0.291
Linear-by-linear Association	0.567	1	0.451
Number of valid cases	102		

a Eleven cells (55 %) have expected count less than 5. The minimum expected count is 0.29.

**Table 6:** Side wise crosstabulation and chi-square test analysis of number of sesamoid bones in each digit.

Digit	Number of sesamoids	Right side (*n* = 51)	Left side (*n* = 51)				
1st	1	3 (5.9 %)	6 (11.8 %)				
	2	28 (54.9 %)	23 (45.1 %)				
	3	20 (39.2 %)	22 (43.1 %)				
	Chi-square tests	Value	df	*p*-value			
	Pearson chi-square	1.585a	2	0.453	a- 2 cells (33.3 %) have expected count less than 5. The minimum expected count is 4.50.		
	likelihood ratio	1.606	2	0.448			
	linear-by-linear association	0.025	1	0.875			
Digit	Number of sesamoids	Right side (*n* = 51)	Left side (*n* = 51)				
2nd	0	37 (72.5 %)	36 (70.6 %)				
	1	13 (25.5 %)	15 (29.4 %)				
	2	1 (2 %)	nil (0 %)				
	Chi-square tests	Value	df	*p*-value			
	Pearson chi-square	1.157a	2	0.561	a- 2 cells (33.3 %) have expected count less than 5. The minimum expected count is 0.50.		
	likelihood ratio	1.543	2	0.462			
	linear-by-linear association	0	1	1			
Digit	Number of sesamoids	Right side (*n* = 51)	Left side (*n* = 51)				
3rd	0	50 (98 %)	50 (98 %)				
	1	1 (2 %)	1 (2 %)				
	Chi-square tests	Value	df	Asymptotic significance (2-sided)	*p*-value	Exact Sig. (1-sided)	
	Pearson chi-square	.000a	1	1			a- 2 cells (50 %) have expected count less than 5. The minimum expected count is 1.00. b- computed only for a 2 × 2 table
	continuity correctionb	0	1	1			
	likelihood ratio	0	1	1			
	Fisher's exact test				1	0.752	
	linear-by-linear association	0	1	1			
Digit	Number of sesamoids	Right side (*n* = 51)	Left side (*n* = 51)				
4th	nil	51 (100 %)	51 (100 %)				
Digit	Number of sesamoids	Right side (*n* = 51)	Left side (*n* = 51)				
5th	0	27 (52.9 %)	26 (51 %)				
	1	24 (47.1 %)	25 (49 %)				
	Chi-square tests	Value	df	Asymptotic significance (2-sided)	*p*-value	Exact Sig. (1-sided)	
	Pearson chi-square	.009a	1	0.925			a- 0 cells (0 %) have expected count less than 5. The minimum expected count is 23.76. b- computed only for a 2 × 2 table
	continuity correctionb	0	1	1			
	likelihood ratio	0.009	1	0.925			
	Fisher's exact test				1	0.542	
	linear-by-linear association	0.009	1	0.925			

## Discussion

The sesamoid bones appear early in embryonic development, as observed in squamate species, which suggests that sesamoids are not merely a result of biomechanical stimuli but may have intrinsic developmental origins.^10^ Genetic predispositions play a role, and environmental factors such as mechanical stress can impact their formation and growth.^11^ The occurrence and nonoccurrence of sesamoid bones in various species and their evolution dictate their adaptive significance. In humans, sesamoid bones are highly prevalent in the thumb and contribute to increased mobility and independence of the digit. In the MCP joints, sesamoids increase the leverage and efficiency of the action of hand muscles.^12^ The ability of the hand to perform complex tasks is due to its biomechanical architecture, which includes the sesamoid bones. The relationship between the distal arm and hand movements affects the magnitude and stability of the grip force, which can impact the sesamoid bones.^13^ The activities that involve repetitive strain on the hand, such as playing musical instruments or sports, can lead to variations in the biomechanical stress impacting the sesamoid bones, which affects their development and function.^14^^,^^15^ Dabrowski et al.^16^ reported that musicians’ hands have larger sesamoid bones than nonmusicians do. The present study investigated the incidence, topography and dimensions of sesamoid bones of the hand in a sample from South India. The sesamoid bone was observed at the first metacarpophalangeal joint, medially and laterally, in 99 % and 80.6 % of the patients, respectively. The second, third and fifth metacarpophalangeal joints exhibited a single sesamoid bone, and they were present in 25.5 %, 3.1 % and 46.9 % of the patients, respectively. The sesamoid bones had a greater incidence in females (*p* < 0.05), and the sidewise, age wise comparison did not yield a statistically significant difference (*p* > 0.05).

Sesamoid bones can be involved in traumatic injuries and can manifest as sesamoiditis, fractures, and degenerative changes. They can be symptomatic because of overuse or injury, which can cause morbidity due to pain and discomfort.^17^^,^^18^ Accurate identification is essential to make an accurate diagnosis and to avoid misinterpretation, such as fractures and degeneration.^18^^,^^19^ Fractures of the sesamoid bones of the thumb are rare and can be easily missed.^20^ Plain X-ray can reveal fracture of a sesamoid on many occasions, but in cases of trauma due to hyperextension involving the MCP joint, ultrasound should be used as an additional investigation. Along with better visualization of fractures, ultrasound can be used to evaluate other structures, such as tendons, ligaments and the volar plate.^21^ In the case of ulnar sesamoid bone fracture, instability of the ulnar collateral ligament or a Stener lesion should be excluded by performing an ultrasound examination on an emergency basis.^20^ Colombo and Shah^22^ reported a case of fracture of the ulnar sesamoid bone of the thumb with a concurrent ulnar collateral ligament tear. The clinician should have a low threshold for advanced imaging in these patients to confirm any suspected ligamentous injury, as the ulnar sesamoid bone of the thumb and the ulnar collateral ligament are in close anatomical proximity. Aslantürk and Ergen^23^ reported a case of a fifth-digit sesamoid fracture, which was treated conservatively. This gave the best result, as the patient had full range of motion of the little finger and hand without the pain observed during the sixth-month follow-up outpatient visit.

Few cases in which tumors such as aneurysmal bone cysts and chondromas involve sesamoid bones and necessitate surgical intervention have been reported.^24^ Arthritis of the sesamoid bones can lead to narrowing of the joint space, sclerosis and bony spurs, which can be misdiagnosed if not properly evaluated via radiographic or sonographic interpretation.^25^ Systemic disorders such as metabolic and hematologic conditions and skeletal dysplasias can involve the hand sesamoid bones, and they have their own characteristic features on hand radiographs.^26^ However, on many occasions, sesamoid bones are only occasionally observed via X-rays and are not associated with symptoms. However, they need to be evaluated carefully to rule out any associated pathology.^17^^,^^27^ These findings suggest that the morphological data of sesamoid bones are important to plastic surgeons, hand surgeons and radiologists.^28^

According to the meta-analysis by Yammine et al.,^29^ the incidence of the sesamoid in the first MCP joint is 99.9 % on the lateral side and 99.8 % medially. In our study, the sesamoid bone was also found on the medial side in 100 % of the patients and on the lateral side in 85.2 % of the patients. Civan et al.^3^ also observed sesamoid bones in the first MCP joint in 100 % of patients bilaterally. Ergen et al.^30^ reported sesamoid bones in the first MCP in 100 % of patients in the Turkish population, and Al Khabori et al.^31^ reported sesamoid bones in 100 % of Omani patients. Amar et al.^8^ reported sesamoids at the first MCP joint in 99.5 % of subjects in the Mediterranean population. Al Khabori et al.^31^ reported sesamoids in the interphalangeal joints of the thumb in 49.7 % of subjects. Interestingly, they reported a greater incidence of sesamoids in females than in males. In the present study, a statistically significant difference was detected between males and females. The females in our samples had a greater incidence (*p* < 0.05), which was also reported by Kose et al.^32^ and Al Khabori et al.^31^ It has been reported that sesamoid bones ossify earlier in females than in males.^33^ The prevalence of interphalangeal joint sesamoid bone of the thumb was 47.1 % in the present study in a sample from the South Indian population. The prevalence is variable globally, as a Turkish study reported 21.3 % prevalence^32^ and 67 % in the Japanese population.^34^ The prevalence of sesamoids in second MCP joints according to Ergen et al.^30^ was 37.6 %, and that according to Al Khabori et al.,^31^ in Omani subjects was 34.6 %. This prevalence was 36.6 % in the Turkish population.^32^ In the present study, the prevalence of sesamoids in the second MCP joint was found to be 25.5 %. Amar et al.^8^ reported a higher frequency of sesamoids in the index finger in 42.3 % of the Mediterranean population. In the present study, a single sesamoid bone in one of the third MCPs was exhibited by only 3.1 % of the patients, which is much less common than in the first two fingers. Dharap et al.^33^ reported a 2.3 % prevalence rate of sesamoids in the third MCP joint in an Arab population. Similar observations were reported by Ergen et al.^30^ In the Turkish population, this was a 1.3 % prevalence rate.^32^ There were no sesamoid bones in the ring finger in the radiographs in the present study from the South Indian population. However, in an Arabian study^33^ this percentage was reported to be 1.5 %, and in the Mediterranean study by Amar et al.,^8^ this percentage was 0.2 %. Kose et al.^32^ observed the sesamoid bone in the fourth MCP joint in 0.9 % of cases. The present study revealed the sesamoid bone in the fifth MCP joint in 47.1 % of the patients. This is similar to previous reports from other populations, 45.3 % in the Arabian population,^33^ 41.1 % in the Mediterranean population^17^ and 53.2 % in the Turkish population.^32^ In the Korean population, there was a higher incidence of sesamoid bones at the MCP joints of the thumb and index finger and the IP joints of the thumb.^35^ Their consistent presence was observed in the thumb MCP joint, with variations in the index and little finger MCP joints in South American and Turkish populations.^9^^,^^32^ A study from Arabians also revealed a higher incidence in the thumb MCP and IP joints.^33^

According to Xu et al.,^36^ the average length of the sesamoid bone of the thumb is 4.46 mm in males and 4.22 mm in females. Notably, in the present study, the length and breadth of the medial sesamoid were 0.49 ± 0.07 cm and 0.40 ± 0.07 cm, respectively. The same dimensions for the lateral sesamoid bone were 0.52 ± 0.10 cm and 0.37 ± 0.08 cm, respectively. In a previous cadaveric anatomical study,^28^ the length, width and thickness of the medial sesamoid of the first MCP joint were 0.3 ± 0.09 cm, 0.62 ± 0.12 cm and 0.48 ± 0.08 cm, respectively. The dimensions of the lateral sesamoid of the first MCP joint in the cadaver were 0.27 ± 0.08 cm, 0.64 ± 0.13 cm and 0.47 ± 0.06 cm. The length and width of the interphalangeal joint sesamoid of the thumb in this study were 0.41 ± 0.1 cm and 0.57 ± 0.12 cm, respectively. In the cadaveric study, the length, width and thickness of this sesamoid were 0.27 ± 0.08, 0.64 ± 0.13 and 0.47 ± 0.06 cm, respectively.^28^ According to Civan et al.,^3^ knowledge of the sesamoid bones of the thumb may be helpful in the diagnosis of certain disorders, such as acromegaly, where the size of the bone is significantly enlarged. The common cause of fracture of the sesamoid bones is hyperextension of the MCP joint or direct compressive injury to the bone. The small surface of the sesamoid bones may cause difficulty in diagnosing fractures. With respect to the thumb, the sub sesamoid joints are the common site for arthritis, and only 80 % of people are symptomatic. A complete radiograph of the patient with subsesamoid arthritis was normal. However, expert evaluation of radiographs may reveal a decreased sub sesamoid space and sclerosis with osteophyte formation.^5^ Considering all these facts, the topography, prevalence and morphometry of sesamoid bones are very important in diagnosing and treating hand disorders. The prevalence of the sesamoid bones compared with that reported in previous studies is consistent with our results. However, the potential limitation of this study was the use of plain radiographs, which might miss few sesamoid bones due to superimposition.^19^^,^^32^ Seki et al.^34^ suggested that lateral viewing radiographs offer better interpretation. However, in the present study, lateral view films were not included, which is another limitation. The evaluation of sesamoid bones with three-dimensional ultrasound, CT, MRI and digital tomosynthesis^19^^,^^32^ could be a better tool to achieve accurate results, which can be a future implication of the present study. It has been reported that digital tomosynthesis is more effective than conventional radiography in detecting sesamoid bones.^19^ Computer tomography and magnetic resonance imaging studies can reveal the different shapes of hand sesamoids, which can be correlated with the occupation of the individual. Radiographs of children, particularly those from five years onward, were not included in this study, which is another major limitation. The study of radiographs in this age group can provide information about the ossification of sesamoid bones, as their appearance and development follow specific age patterns. However, this study considered the age group of fourteen years onward because the ossification of sesamoid bones in the hand, particularly at the thumb MCP joint, generally occurs around the age of fourteen years.^33^ This process is closely linked to the onset and progression of puberty, making it a useful marker for assessing skeletal maturity.^37^ Future implications also include studying the role of sesamoid bones in contributing to hand function and understanding how their absence, presence or variation impacts hand mechanics. Artificial intelligence^38^-powered diagnostic systems can enhance clinical examination, diagnosis, and treatment by analyzing vast datasets, improving accuracy, and detecting subtle anomalies such as variations in sesamoid bones. By enhancing diagnostic accuracy, enabling perfect surgical planning, and supporting personalized treatment strategies, AI can significantly improve health outcomes.^39^ However, AI technologies such as the ChatGPT have drawbacks, as they provide insufficient anatomical information to clinicians, particularly for smaller structures such as sesamoid bones.^40^ The novelty of the present study is that it offers the morphometric data of the sesamoid bone of each digit separately. The length and width of the sesamoid bones of the hand, particularly those of the index finger, middle finger and little finger, are not reported in the literature.

## Conclusion

The prevalence of the sesamoid bone in the first MCP is greater than that in the other digits. The sesamoid bones were more common in females than in males. The prevalence, topography and morphometric data of the sesamoid bones of the hand reported in this study can be used as a morphological database for the South Indian population. The data will help operating hand surgeons and radiologists in their clinical practice.

## Ethical statement

The authors state that every effort was made to follow their institutional and international ethical guidelines and laws that pertain to this research. This study was approved by the ethics committee of our institution.

## Funding

This research did not receive any specific grant from funding agencies in the public, commercial, or not-for-profit sectors.

## Patient consent statement

Since the identity of patients is not revealed in any of the sections of this manuscript, including the figures, written informed consent from the patients is not needed.

## CRediT authorship contribution statement

**Phajir V. Santosh Rai:** Supervision, Validation. **Ravichandraprabhu Abisshek Balaji:** Data curation, Formal analysis, Investigation. **Madhvi Yadav:** Data curation. **Jefferson Prince:** Writing – review & editing. **Latha V. Prabhu:** Writing – review & editing. **Rohini Punja:** Writing – review & editing. **Mamatha Hosapatna:** Writing – review & editing. **Bukkambudhi V. Murlimanju:** Conceptualization, Data curation, Formal analysis, Investigation, Methodology, Validation, Visualization, Writing – original draft.

## Declaration of competing interest

None declared.
